# Principal Component Analysis Enhanced with Bootstrapped Confidence Interval for the Classification of Parkinsonian Patients Using Gaussian Mixture Model and Gait Initiation Parameters

**DOI:** 10.3390/s24061885

**Published:** 2024-03-15

**Authors:** Florent Loete, Arnaud Simonet, Paul Fourcade, Eric Yiou, Arnaud Delafontaine

**Affiliations:** 1 Laboratoire de Génie Électrique et Électronique de Paris, CNRS, Centrale Supélec, Université Paris-Saclay, 91190 Gif-sur-Yvette, France; 2 CIAMS, Université Paris-Saclay, 91405 Orsay, France; simonet.arnaud@ladapt.net (A.S.);; 3 CIAMS, Université d’Orléans, 45067 Orléans, France; 4 Laboratoire D’Anatomie Fonctionnelle, Faculté des Sciences de la Motricité, Route de Lennik 808, Université Libre de Bruxelles, CP 619-1070 Bruxelles, Belgium; 5 Laboratoire d’Anatomie, de Biomécanique et d’Organogenèse, Faculté de Médecine, Route de Lennik 808, Université Libre de Bruxelles, CP 619-1070 Bruxelles, Belgium

**Keywords:** gait initiation, Parkinson’s disease, principal component analysis, bootstrapping, rehabilitation, unsupervised learning

## Abstract

Parkinson’s disease is one of the major neurodegenerative diseases that affects the postural stability of patients, especially during gait initiation. There is actually an increasing demand for the development of new non-pharmacological tools that can easily classify healthy/affected patients as well as the degree of evolution of the disease. The experimental characterization of gait initiation (GI) is usually done through the simultaneous acquisition of about 20 variables, resulting in very large datasets. Dimension reduction tools are therefore suitable, considering the complexity of the physiological processes involved. The principal Component Analysis (PCA) is very powerful at reducing the dimensionality of large datasets and emphasizing correlations between variables. In this paper, the Principal Component Analysis (PCA) was enhanced with bootstrapping and applied to the study of the GI to identify the 3 majors sets of variables influencing the postural control disability of Parkinsonian patients during GI. We show that the combination of these methods can lead to a significant improvement in the unsupervised classification of healthy/affected patients using a Gaussian mixture model, since it leads to a reduced confidence interval on the estimated parameters. The benefits of this method for the identification and study of the efficiency of potential treatments is not addressed in this paper but could be addressed in future works.

## 1. Introduction

Epidemiological studies reported that Parkinson’s disease (PD) is the second world-wide neurodegenerative pathology. In 2016, 6.1 million people were affected by PD [1,2]. PD is anatomically characterized by an alteration of the basal ganglia resulting in a dopamine deficiency clinically responsible for both cognitive and motor disorders, including postural instability [3] and a difficulty to initiate voluntary movements such as gait [4]. In more severe cases, the instability can lead to gait freezing or falling [5].

The heterogeneity of PD symptoms involves the fact that patients are taken in charge through a multidisciplinary team approach combining complementary treatments [1], typically pharmacological (Levodopa intake being the “first line” of medication [6]), neurosurgical (e.g., deep brain stimulation [7]), or non-pharmacological rehabilitation treatments [8,9,10]. However, as stressed in the literature, there is currently a need to provide new non-pharmaceutical protocols to classify the progression of the disease and evaluate the efficiency of these treatments [11].

The biomechanical analysis of gait initiation (GI), which is a main issue in PD patients, is relevant to provide information on the progression of the disease and on the efficiency of these treatments [12]. For example, two studies have recently shown the efficiency of non-pharmacological rehabilitation interventions on the improvement of GI in PD patients. They were based on either acute triceps surae (TS) stretching [13] or functional electrical stimulation bilaterally applied to tibialis anterior [14].

GI corresponds to the transient period between the quiet standing posture and steady-state walking [15]. It is composed of two successive phases: the “postural phase”, where the so-called “anticipatory postural adjustments” (APAs) are developed, precedes the swing foot-off and the “step execution phase” [16]. The biomechanical characterization of GI is usually done through the simultaneous acquisition of a compound of kinetic variables resulting in very large datasets. A typical dataset could reach up to around 20 parameters including [17]:

- The mediolateral (ML)/anteroposterior (AP) APA
- The center of pressure (COP) shift/velocity AP/ML
- The center of mass (COM) shift/velocity AP/ML at heel off (HO), toe off (TO) and heel contact (HC)
- The foot-lift: time between HO and TO
- The step execution time (EXE)
- The vertical peak force at HC

Those high dimensional datasets are difficult to analyze, since the time-dependent kinetic variables are highly correlated [18].

The principal component analysis (PCA), which is a powerful unsupervised machine learning technique, reduces the dimensionality of large datasets while preserving the maximum information (variance). It is consequently able to identify the hidden correlations between the parameters in very complex, high-dimensional datasets. The work is conducted through a single computation, which is much more convenient than conducting a classical univariate analysis [13,19]. It was applied in the clinical field to investigate the influence of various joint pathologies such as knee osteoarthritis [20] or to characterize the biomechanical GI differences between elderly fallers and non-fallers [21]. The ability of the technique to accurately discriminate normal and abnormal gait was also evidenced in [22]. The PCA was also used to analyze the effect of subthalamic stimulation [23] on PD patients or to create a model of hip and knee joint-angular velocity from limb kinematic data [24].

In theoretical statistics, the central limit theorem (CLT) states that if the sample size is large, then the average is normally distributed. In practice, it is difficult, especially in the medical field, to gather large enough sample data on patients to consider the CLT. Consequently the statistical analysis is often conducted using the mean and the confidence interval of the samples data. The bootstrapping method can be used to construct a confidence interval of the mean from a very small dataset in a sense that it models the population by resampling the sample data. This confidence interval should be smaller than the sample one; consequently, this method could be more relevant than the traditional univariate ones.

PCA combined with bootstrapping has been successfully applied in various fields including gene identification or the cognitive-network study with Alzheimer disease [25,26]. In the present paper, we investigate the benefits of combining PCA and bootstrapping for the identification of biomechanical GI markers on PD patients and the development of an efficient classification methodology of the disease status.

While in [27], PCA and classification approaches such as SVM, PNN and linear regression were applied to raw ground reaction force recordings, in this paper this method is described for the first time on a more complete set of variables extracted from the ground force measurement and achieving a very effective classification, using a Gaussian mixture model, of a population of healthy and Parkinsonian patients.

## 2. Methods

### 2.1. Principal Component Analysis and Bootstrapping

**Figure 1:**
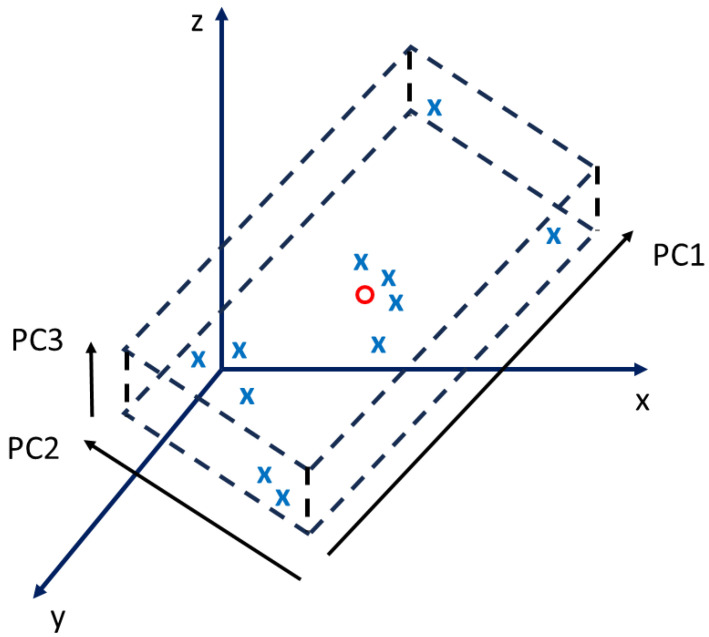

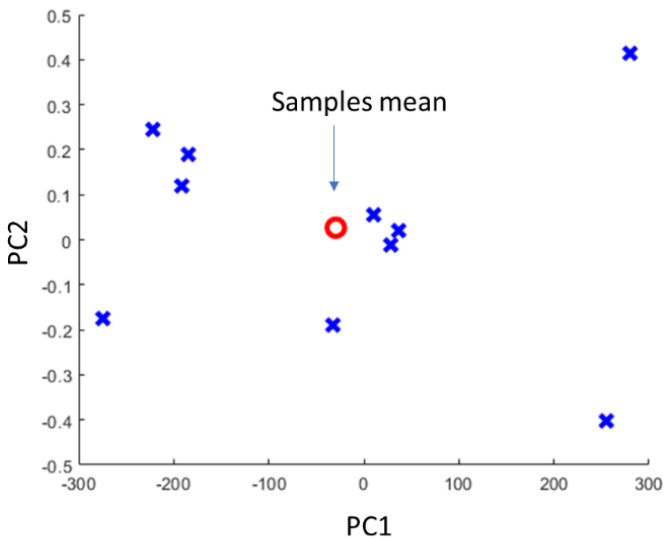
Scatter plot of the scores of a 3D point cloud of samples (**x**) and their mean (O) into the subspace of the first two principal components PC1 and PC2. The projection into the 2D subspace minimizes the information loss.

It is not the purpose of this work to delve into the details of the mathematical method, but the basic principles are recalled here for clarity. The principal components are uncorrelated, linear combinations, from one or several variables of the initial dataset. In a multidimensional space, they are the directions that explain the most variance; therefore, projecting the dataset on these vectors will minimize the information loss. The correlations between the features can also be determined by looking at the linear combination of variables within the principal components. The power of the PCA is its ability to identify, in a large raw dataset, the parameters of interest and their correlations in a single, very fast computation. A simple example for illustration purposes is provided in Figure 1, in which a point cloud is located in a shallow region of the space. The dimension that explains little variance can therefore be neglected, since it provides little information. The PCA finds the principal components (black arrows), and the point cloud can be projected in the subspace of the two most significant ones. PC1 is a linear combination of y-z, while PC2 is a linear combination of x-z. y-z and x-z are, respectively, positively and negatively correlated.

### 2.2. Bootstrapping

**Figure 2:**
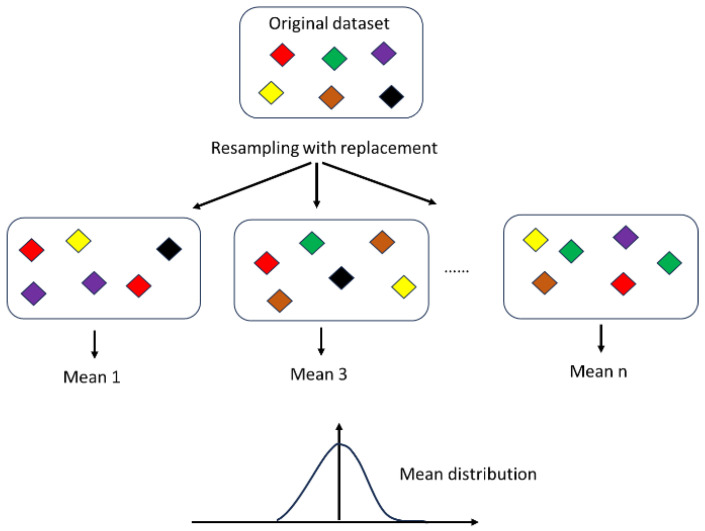
Bootstrapping principle: statistical method that allows one to estimate the distribution of a parameter inside the population using sampling with replacement.

The traditional hypothesis testing uses the obtained dataset to calculate various statistics, such as the mean or the standard deviation, in order to draw inferences on the population. The central limit theorem states that if the sample size is large enough, then the mean of a random sample from a population will distribute normally, regardless of the underlying distribution. A minimum sample size of 30 is usually considered. Repeating the study or having a large sample size is not always easily feasible in the medical field and especially in GI investigation, since it is usually very difficult to gather enough patients. In this paper, the bootstrapping procedure, which is a resampling technique with replacement, was used to construct a confidence interval for the calculated mean on our small dataset. This involves repeatedly drawing samples from our dataset in order to generate multiple new simulated datasets of the same size as the original. Each simulated dataset has its own properties, and calculating the mean of each would yield a distribution of the means (Figure 2). The process can be repeated as many times as desired to obtain a robust statistical estimation.

**Figure 3:**
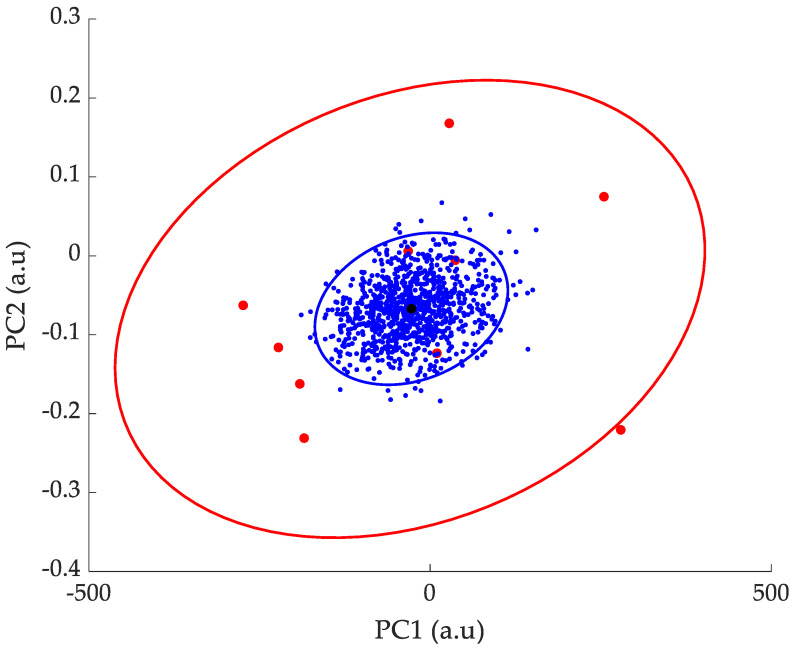
Using a limited number of samples (●) with mean (●) and a 95% confidence interval (**-**), the bootstrapping method allows one to calculate 1000 bootstrapped means (●) with a reduced 95% confidence interval (**-**).

In the case of a real test including 20 patients, 1000 bootstraps would be equivalent to 1000 different tests from 20 patients. Figure 3 shows how the 1000 bootstrapped means are distributed around the one from the original sample dataset. Bootstrapping will converge on the correct distribution with a sample size as low as 10. This method is very useful when access is limited to a very small number of individuals inside the population. The 95% confidence interval of the means shown in Figure 3 can be easily obtained from the covariance matrix. Very interestingly, the confidence interval of the sample mean is much reduced compared to the one of the original samples. In Section 3, we will demonstrate how this can be purposefully used to enhance the classification of two groups of patients (healthy and Parkinsonian).

### 2.3. Participants and Sample Collection

In this paper, a set of previously published GI kinetics data from PD patients and a control group was purposely used [13]. Ten Parkinson-disease-affected patients were recruited for this study. The “control group” was composed of ten healthy, community-dwelling seniors, of the same age and walking without assistance. They were free of any known neuromuscular disorders. In this study, they provided normative values of dependent variables. PD patients were examined by a neuro-physiotherapist after a 12 h withdrawal from antiparkinson’s medications (here referred to as the OFF-medication state). The examination was based on the Movement Disorder Society Clinical Diagnostic Criteria for Parkinson’s disease [28]. PD patients had no known records of falling or freezing. They were also physically perfectly independent and able to walk without any assistance. The participants were required to give written informed consent after being informed about the nature and purpose of the experiment. The protocol, in compliance with the standards established by the Declaration of Helsinki, was previously approved by the local ethics committee of the University of Paris-Saclay and registered under the following trial number: 2018-A00162–53.

### 2.4. Experimental Task

**Figure 4:**
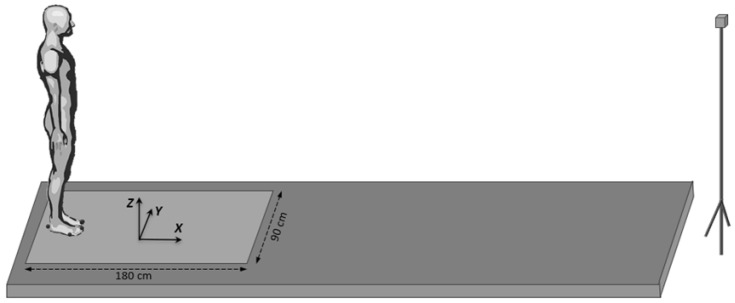
Experimental setup used for acquiring the gait initiation data on a force-plate.

The participants stood at first in a natural upright position on a force-plate (0.9 × 1.80 m, AMTI, Watertown, MA, USA), which was located at the beginning of a 6 m walkway track (Figure 4). The force-plate recorded the 3D forces and moments acting at its surface. In the initial standing posture, the feet were positioned shoulder-width apart, the arms rested alongside the trunk, and the gaze was directed forward to a small target at eye level (2 cm diameter, 6 m away). The locations of the heel and big toe of each foot were marked on the force-plate with strips of adhesive tape and were used as a visual reference on which the participants positioned themselves after each trial under the supervision of the experimenters.

Two series of ten barefoot gait initiations were performed. When instructed, they initiated the gait, with a prior “all set” signal, at a spontaneous velocity when they felt ready. The participants’ preferred leg was determined by lightly pushing on the participants’ backs in the initial posture, with eyes closed, to induce a step forward. The walking movement was continued until the end of the track, and they were required to rest ~10 s between trials.

### 2.5. Data Recordings of the Experimental Variables

**Figure 5:**
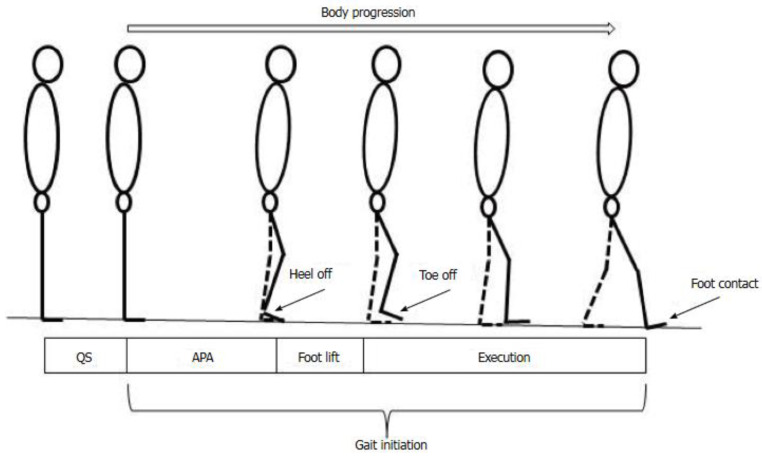
Schematic representation of the different phases and temporal events of the gait initiation.

The characteristic time-events were extracted from the biomechanical traces: GI onset (t0), swing heel-off, swing foot-off and swing foot-contact [17] (Figure 5).

GI is divided into APA (from t0 to HO), foot-lift (from HO to TO) and execution phase (from TO to foot-contact (FC) [15]). APA amplitude was characterized by the maximal mediolateral and anteroposterior COP displacement obtained during the APA time-window and the COM velocity at swing heel-off.

In this paper, we chose to build our dataset using the following 11 experimental variables, as they have proven to be sufficient to properly describe the GI process [17] and can be easily derived from the force plate data:

- The ML/AP APA (in *s*): ${APA}_{ML}$ and ${APA}_{AP}$
- The AP/ML COP shift (in *m*)
- The AP/ML COM velocity at HO, TO and HC (in *m*/*s*)
- The foot-lift time between HO and TO (in *s*)
- The vertical peak force at HC (in *N*)

The instantaneous COM acceleration was derived directly from the recorded ground reaction forces and the application of Newton’s second law. The COM velocity was computed by numerical integrations of the COM acceleration.

The data were acquired at a sampling rate of 500 Hz and filtered with a second-order Butterworth low-pass filter designed with a 10 Hz cut-off frequency [29]. The data acquisition was controlled by a custom-made program written in Matlab^TM^ (Version 5.3 (R11), The MathWorks Inc., USA).

## 3. Results on the Classification of Healthy and Parkinsonian Patients

In this section, we were interested in whether the PCA enhanced with bootstrapping could help to classify healthy and affected Parkinsonian patients. The GI dataset was run through PCA, and the mean scores were calculated and bootstrapped 10,000 times. It was found that the three first principal components, provided in Equation (1), explain 99.9% of the variance, thus reducing the 11 dimensions to only 3:

(1) $$\begin{array}{c} PC1=F_{z}\\ PC2=0.71*{APA}_{ML}+0.56*{APA}_{AP}-0.41*V_{CG_{AP}@HC}\\ PC3=0.791*V_{CG_{AP}@HC}+0.25*{APA}_{ML}+0.28*{APA}_{AP}+0.27*V_{CG_{AP}@HO}+0.37*V_{CG_{AP}@TO} \end{array}$$

where $F_{z}$, $V_{CG_{AP}@HC}$, $V_{CG_{AP}@HO}$, $V_{CG_{AP}@TO},\;{APA}_{ML}$ and ${APA}_{AP}$ are, respectively, the peak force at HC, the AP COM velocity at HC, HO, TO and the ML/AP APA.

The linear combinations PC1, PC2 and PC3 of those variables are consequently the ones that retain more information from the original dataset of 11 dimensions. This is consequently the best subspace in which to project and observe the samples.

One can note in PC2 and PC3 that the coefficients affected to ${APA}_{ML}$ and ${APA}_{AP}$ are similar; thus, ${APA}_{ML}$ and ${APA}_{AP}$ could be replaced by ${APA}_{Mean}$, the mean APA time. In the same way, $V_{CG_{AP}@HO}$ and $V_{CG_{AP}@TO}$ could quite logically be replaced by $V_{CG_{AP}@HO}$ only, since there is not much difference between the COM velocity of HO and TO. The main variables allowing one to classify the two different groups are consequently the peak force at HC, the AP COM velocity at HO and HC, as well as the mean APA.

The principal components can therefore be simplified as follows:

(2) $$\begin{array}{c} PC1=F_{z}\\ PC2\approx {APA}_{mean}-0.41*V_{CG_{AP}@HC}\\ PC3\approx 0.791*V_{CG_{AP}@HC}+0.5*{APA}_{mean}+0.64*V_{CG_{AP}@HO} \end{array}$$

**Figure 6:**
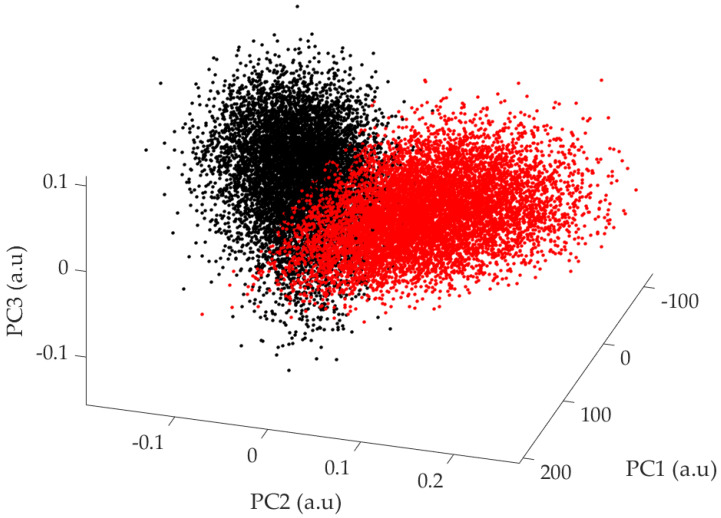
Comparative plot of 10,000 bootstrapped means of the PCA scores of the control group (●) and the Parkinsonian patients (●) on the PC1/PC2/PC3 subspace.

**Figure 7:**
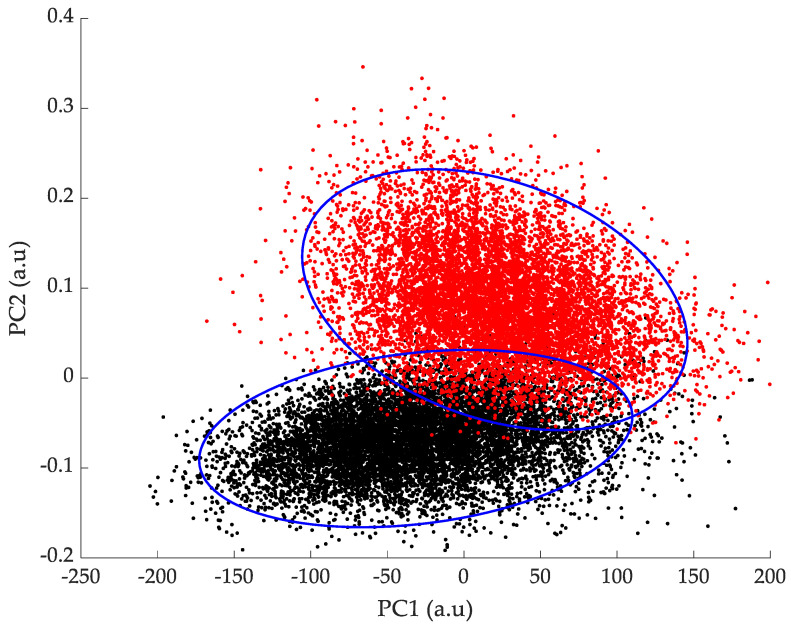

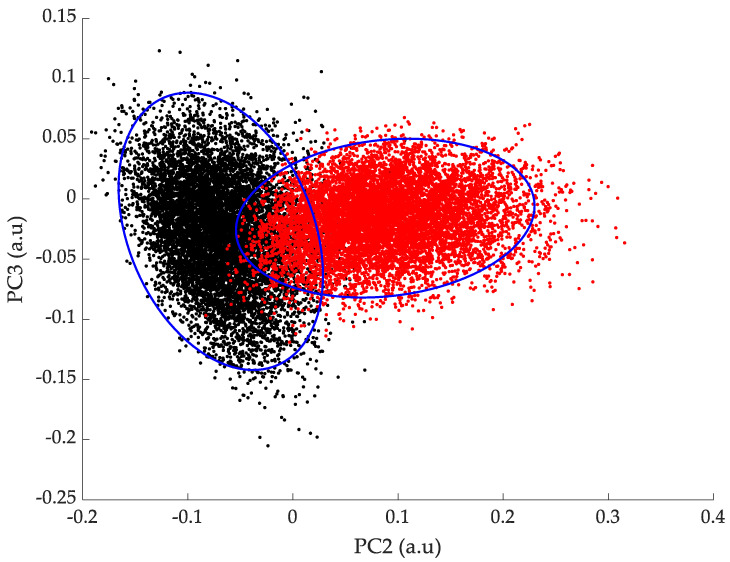
2D plots of 10,000 bootstrapped means of the PCA scores of the (●) control group and (●) Parkinsonian patients in (**top**) the PC1/PC2 subspace and (**bottom**) the PC2/PC3 subspace. The ellipses (**-**) represent the 95% confidence intervals.

In Figure 6, 10,000 bootstrapped means for both the control group and the PD patients are plotted in the 3D subspace of the first three principal components. The two 3D point clouds appear well separated, but in order to better visualize the cluster separation, Figure 7 shows the projections on the 2D subspaces PC1/PC2 and PC2/PC3.

In Figure 6, the clusters corresponding to the healthy and Parkinsonian patients can be clearly visually discriminated. In order to quantify their degree of separation, the Jaccard similarity coefficient, which provides a robust measure for assessing the degree of overlap and commonality within distinct datasets, was calculated. Specifically designed for finite sample sets, this coefficient calculates the similarity between two datasets A and B by determining the ratio of the size of the intersection to the size of the union of the sample sets:

(3) $$J\left(A,B\right)=\frac{\left|A\cap B\right|}{\left|A\cup B\right|}$$

The Jaccard similarity coefficient $J\left(A,B\right)$ can thus be regarded as the probability of picking an element present in both sets.

In the PC1/PC2 and the PC2/PC3 subspaces, the Jaccard coefficients were, respectively, 10.9% and 11.2%. This result emphasizes the ability of the PCA combined with bootstrapping to accurately identify and differentiate two clusters corresponding to a healthy and a Parkinsonian population. The addition of bootstrapping to PCA significantly enhances the selectivity of the method, since, as shown in Figure 3, the 95% confidence interval of the data samples alone would be much larger than the one of the bootstrapped means, resulting in a higher overlap and Jaccard coefficients.

## 4. Unsupervised Clustering Using Gaussian Mixture Model

In this section, we show how by using unsupervised clustering on the results presented in Section 3, one can classify the status of a patient without any prior knowledge of their status. Unsupervised clustering is a powerful tool for analyzing complex datasets without the need for labeled information and for grouping the data points into clusters based on their similarity. The Gaussian Mixture Model (GMM), which is an expectation-Maximization algorithm, assumes that the data points are a combination of several Gaussian normal distributions. It can be regarded as a generalized version of the classical K-Means algorithm, taking into account the covariance of the data and enabling a more flexible and nuanced representation of cluster boundaries.

The rich statistical framework of GMM-based clustering and its inherent ability to capture the uncertainty in data is particularly useful for tasks such as anomaly detection and is particularly well-suited for modeling complex datasets with overlapping or irregularly shaped clusters.

**Figure 8:**
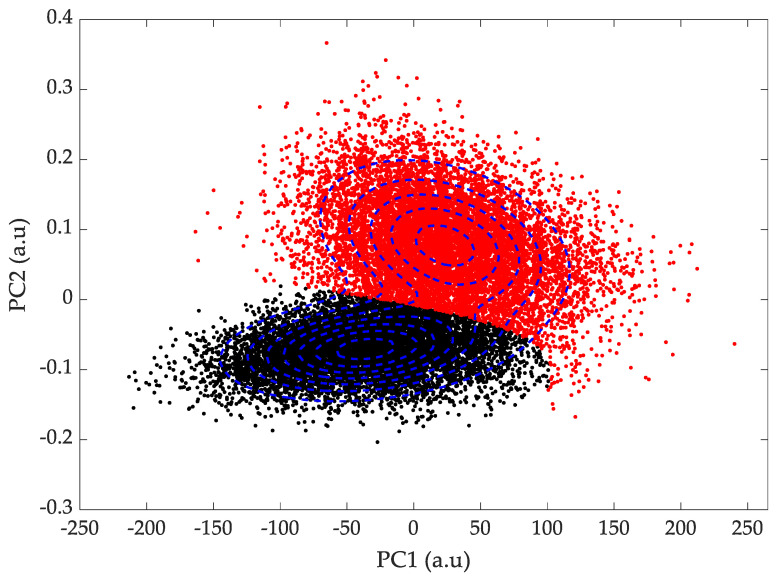

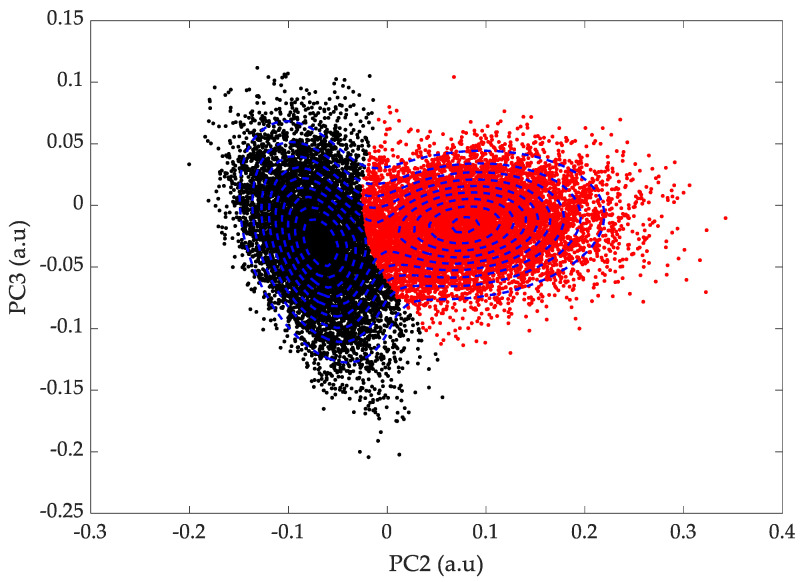
Clustering of 20,000 bootstrapped means using Gaussian Mixture Model of healthy and Parkinsonian patients using gait parameters. (●) Control group and (●) Parkinsonian patients in (**top**) the PC1/PC2 subspace and (**bottom**) the PC2/PC3 subspace. (**- -**) Contour plot of the GMM distribution.

Since in our case the bootstrapped means fit a Gaussian distribution, this approach is perfectly suited to our datasets. In Figure 8, the GMM clustering was applied to 20,000 bootstrapped means of healthy and Parkinsonian patients in the PC1/PC2 and PC2/PC3 subspaces to see how the means were classified. The algorithm very satisfyingly manages to classify healthy and Parkinsonian patients without prior knowledge of their state, as shown by the confusion matrix shown in Table 1 and Table 2. Parkinsonian patients are properly classified with an accuracy of 98% in both subspaces.

## 5. Conclusions

Considering the complexity of the GI characterization and the need to develop new non-pharmacological tools to classify patients and evaluate the progression of the disease, e.g., [13,17], in this paper we studied the ability of the Principal Component Analysis (PCA) enhanced with bootstrapping to reduce the dimensionality of the dataset, with the aim to classify patients using a Gaussian Mixture Model (GMM). The PCA is a statistical tool that has been shown to be efficient in reducing the dimensionality of datasets [30]. It can rapidly identify the major linear combinations of variables influencing the studied phenomenon. While in [27] PCA and a few classification approaches were tested successfully, they were only applied to raw ground reaction force recordings. In this paper, the results emphasized that three majors sets composed of linear combinations of the peak force at HC, the AP COM velocity at HO and HC, as well as the mean APA are sufficient to classify the status of a patient. The bootstrapping, which consists in resampling the dataset with a replacement, applied to the mean of the PCA scores, was shown to estimate much narrower confidence intervals, thus resulting in an enhanced separation of the clusters. Finally, the GMM was shown to be an efficient clustering method, using only two of the three first principal components.

It is worth noting that in Parkinson’s disease, the evolution of the symptoms, their nature (e.g., some patients show a freezing behavior during gait initiation, while others do not), the age at which they first appear (Parkinson’s disease generally begins at the age of 60 years but might occasionally begin before 50 years and even at the age of 25 years), etc. are highly variable between patients, which makes this disease unique and difficult to diagnose [31]. The present study shows that this methodology comes in handy for clinicians by allowing them to estimate a sampling variability and thus better take into consideration the heterogeneity between patients. Such heterogeneity could not be addressed in previous researches focusing on the biomechanical organization of gait initiation in patients with Parkinson’s disease, as only univariate analyses were carried out, e.g., [32,33,34,35]. The gait initiation analysis proposed in the present study is thus very likely more relevant and robust for detecting Parkinson’s disease than the latter analyses. It is believed that it might beneficially be extended to the detection of other neurological diseases with postural impairments, such as multiple sclerosis, stroke, cerebral palsy, etc.

In conclusion, the application of the proposed unsupervised learning methods offers a new insight into the analysis of gait kinetics and the efficiency of non-drug interventions for physical medicine and rehabilitation clinicians as well as biomedical engineers. The blind identification on a large dataset of unsorted variables of the main parameters evolving with the disease could help physicians assess the way the status of a patient is evolving and could help engineers develop new follow-up tools.

## Figures and Tables

**Table 1:** Confusion matrix used for classification in the PC1/PC2 subspace using a GMM.

	Healthy	Parkinsonian
Healthy	89.8% True healthy	10.2% False Parkinsonian
Parkinsonian	1.9% False healthy	98.1% True Parkinsonian

**Table 2:** Confusion matrix used for classification in the PC2/PC3 subspace using a GMM.

	Healthy	Parkinsonian
Healthy	92% True healthy	8% False Parkinsonian
Parkinsonian	2.5% False healthy	97.5% True Parkinsonian

## Data Availability

Data is contained within the article.
